# Trauma- and Violence-Informed Care: Orienting Intimate Partner Violence Interventions to Equity

**DOI:** 10.1007/s40471-022-00307-7

**Published:** 2022-10-03

**Authors:** C. Nadine Wathen, Tara Mantler

**Affiliations:** 1 Arthur Labatt Family School of Nursing, Western University, FIMS & Nursing Building, Room 2307, London, ON N6A 5B9 Canada; 2 School of Health Studies, Western University, London, Canada

**Keywords:** Intimate partner violence, Structural violence, Health equity, Trauma-informed practice, Trauma- and violence-informed care, Intervention research

## Abstract

**Purposeof Review:**

Intimate partner violence (IPV) is a complex traumatic experience that often co-occurs, or is causally linked, with other forms of structural violence and oppression. However, few IPV interventions integrate this social-ecological perspective. We examine trauma- and violence-informed care (TVIC) in the context of existing IPV interventions as an explicitly equity-oriented approach to IPV prevention and response.

**Recent Findings:**

Systematic reviews of IPV interventions along the public health prevention spectrum show mixed findings, with those with a theoretically grounded, structural approach that integrates a trauma lens more likely to show benefit.

**Summary:**

TVIC, embedded in survivor-centered protocols with an explicit theory of change, is emerging as an equity-promoting approach underpinning IPV intervention. Explicit attention to structural violence and the complexity of IPV, systems and sites of intervention, and survivors’ diverse and intersectional lived experiences has significant potential to transform policy and practice.

## Introduction

Intimate partner violence (IPV), defined as “behaviour within an intimate relationship that causes or has the potential to cause physical, sexual, or psychological harm, including acts of physical aggression, sexual coercion, psychological abuse, and controlling behaviours” (World Health Organization (WHO)) [1], is a major public health crisis [2], with global data from 2018 indicating that 27% of women experience IPV in their lifetime, and 13% in the past year [3•]. IPV, and responses to it, has been made significantly worse by the COVID-19 pandemic [4, 5] with multiple pre- and co-existing pandemics coming together to further exacerbate both prevalence and incidence of IPV, and the ability to balance adequate service responses for survivors with pandemic restrictions intended to curb disease spread [6]. The health and social consequences of IPV on survivors, especially women, and on families and society are well-documented, including worse physical and mental health [7], increased health risk behaviors [8, 9, 10], greater harm to children exposed to IPV [11, 12], and significant costs to health and social services, and entire economies [13•, 14].

### Conceptualizing IPV

**Fig. 1:**
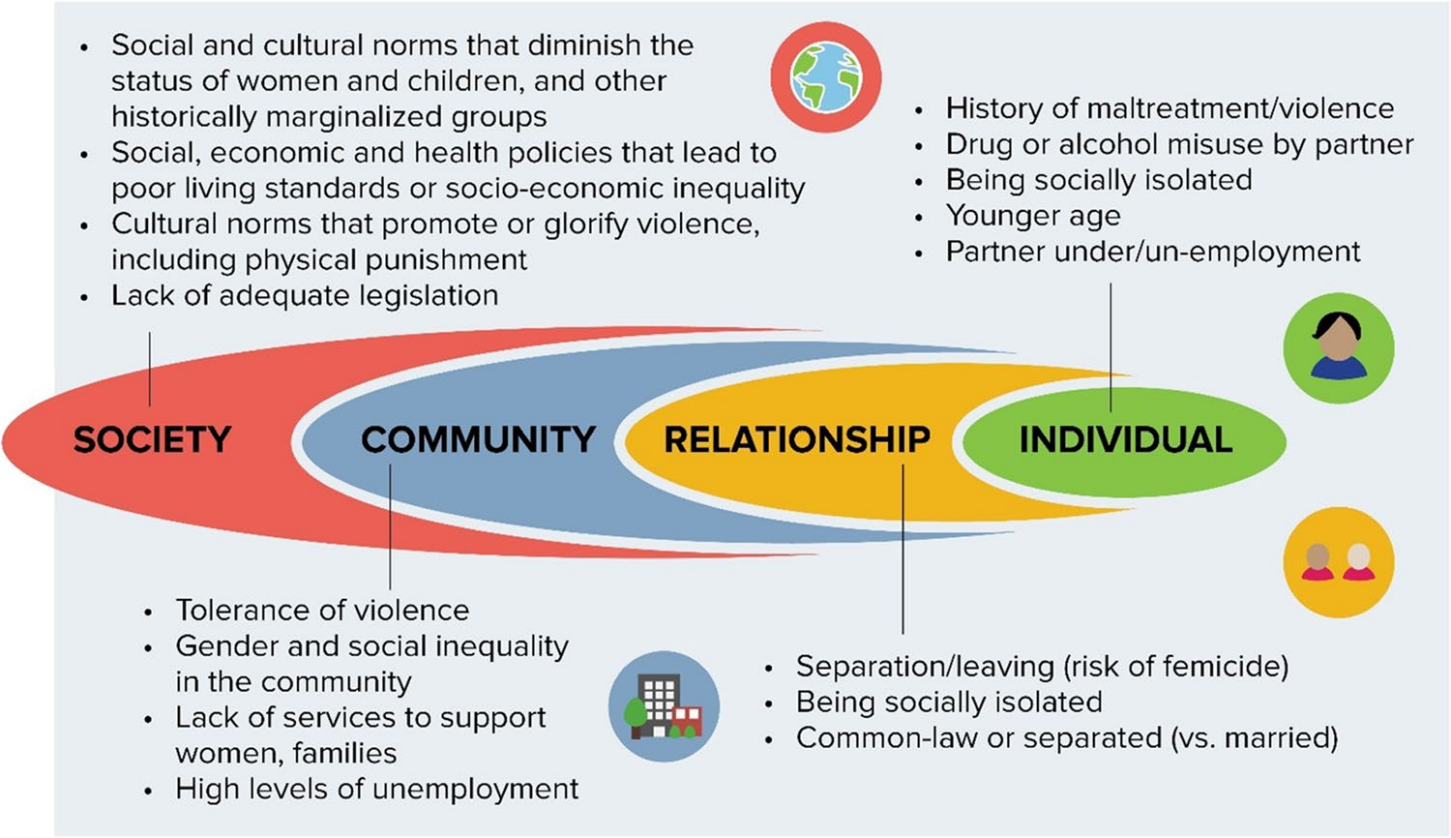
WHO-VPA Ecological Model, adapted from [16]

The past 25 years have seen a significant increase in development and evaluation of interventions for those experiencing IPV, and innovations in measurement and framing of IPV are providing a much-enhanced picture of the epidemiology of this complex social issue. The Violence Prevention Alliance of the WHO promotes the use of an ecological model within the public health approach [15•, 16, 17], based on evidence that no single factor can explain why some people or groups are at higher risk of violence and its consequences, while others are more protected from it. This framework views IPV as the outcome of interactions among many factors and structures at individual, relationship, community, and societal levels, which create both the conditions for IPV, and potential intervention sites across the prevention spectrum. Importantly, this structural approach means that these conditions are viewed as arising from, and rooted in, the values, attitudes, and beliefs that we, collectively, have internalized, and that are embedded in our organizations and communities and reflected and reified by society (Fig. 1).

Similarly, recent advances in measurement of IPV indicate that focusing on the more severe forms of violence rather than less severe, situational, and often bi-directional aggressive behaviors [18], while also attending to the context in which violence occurs, avoids what has been termed the “de-gendering” of IPV [19]. Use of measures that recognize IPV as gendered and patterned [20•, 21•] has helped us connect these experiences to related causes and consequences across the ecological framework and support the development of interventions that account for the complexities of people’s personal and social lives.

### Bringing a Trauma Lens: Trauma-Informed Practice

Trauma is both the experience of and response to an overwhelmingly negative event or series of events, from wars and disasters to accidents and loss (e.g., of a parent) [22]. Events become traumatic due to complex interactions among someone’s neurobiology (affecting, for example, their ability to self-regulate), and their previous experiences of trauma and violence, including the role (or not) of supportive individuals and communities in self-regulation and recovery. Trauma can change brain and nervous system functioning, and while these neurobiological changes may not be permanent, they can be long-lasting, and impact behavior [23, 24]. For example, adverse childhood experiences (ACEs), especially maltreatment, neglect, and experiencing IPV in the family, can have long-term effects including stress, anxiety, depression, risky behaviors, and substance misuse [25, 26, 27]. Complex trauma can also impact child development, leading to internalizing, externalizing, and attachment disorders [28], which can persist into adulthood. Experiencing violence can change not only neurobiological patterns, but also genetic structures [29, 30•], leading to impacts on health and well-being [31]. Thus, exposure to trauma and violence can have long-term effects on health and behavior, whether trauma is ongoing or in the past, individual or collective [32, 33•]. In the context of IPV, trauma can be acute (resulting from a single event) or more likely, complex and chronic.

When serving IPV survivors, providers, organizations, and systems lacking understanding of its complex and lasting impacts miss opportunities to provide effective services, and risk causing further harm. This growing understanding has led to an integration of trauma awareness into services for IPV survivors. Work from the US Substance Abuse and Mental Health Administration [34] specific to women, violence, and substance use helped establish the concept of “trauma-informed practice” (TIP). TIP aims to create safety for people seeking care by understanding the effects of trauma, and its close links to health and behavior. Unlike trauma-specific care, it is not about eliciting or treating people’s trauma histories but about creating safe spaces that limit the potential for further harm in care interactions [22, 35, 36, 37, 38].

However, with some exceptions that take an explicitly organizational approach [39, 40], the focus of TIP is individual, which tends to reinforce the idea that the effects of trauma are located in the individual. Such a focus often leads to strategies to identify “what’s wrong” with a person, so as to avoid doing additional harm, and intervention is located in an individual provider, not a team, organization, or system. By extension, interventions then focus on the individual and their trauma experiences, rather than the factors that shape and even enable those experiences and present barriers to prevention and care, making it more difficult to meet survivors’ complex needs [41, 42]. For example, a recent review of measures in this area found that few TIP approaches truly address structural forms of violence, including stigmatizing and discriminatory beliefs and practices [43•].

### An Equity Perspective: from TIP to TVIC

Approaches to addressing violence in health and social care are beginning to evolve from this narrow focus on interventions for/by individuals to a broader understanding of IPV and other forms of gender-based violence as pervasive social problems embedded in structural inequities. This means explicitly linking interpersonal violence with the broader conditions of people’s lives, including their access to social determinants of health, and their experiences of various intersecting forms of structural violence, including policy-induced violence such as systemic poverty and homelessness (due, for example, to minimum wage legislation and housing policies) and socially induced violence, including racism and other forms of discrimination [44, 45, 46]. As the WHO’s ecological framework highlights, sexism, misogyny, and gender norms are specific root causes of IPV. Seen intersectionally, it is not surprising that worldwide data indicate that specific groups (women, especially those who are racialized, Indigenous, disabled, and/or poor) are over-represented in both prevalence data, and bear the greatest health and social impacts of violence, including barriers to services, income supports, and safe housing [3•].

Trauma- and violence-informed care (TVIC) expands the concept of TIP to account for these intersecting impacts of systemic and interpersonal violence and structural inequities on a person’s life. The four principles of TVIC are as follows: (1) understanding and awareness of trauma and violence, especially structural violence, and their impacts on people’s lives; (2) prioritizing people’s (including providers’) physical, emotional, and cultural safety; (3) promoting person-centered connection, collaboration, and choice; and (4) finding and building on people’s existing strengths, and supporting their skills and capacity development [47••]. This shift is important as it emphasizes both historical and ongoing violence and their traumatic impacts and focuses on a person’s experiences of past and current violence such that problems are seen as residing in both their psychological state and their social circumstances [47••, 48]. TVIC also attends to systemic and institutional violence, including policies and practices that perpetuate harm to satisfy the needs of the system, rather than those of the person (for example, people often have to formally disclose IPV, and then re-tell their experiences to multiple care providers to receive services). TVIC also prioritizes the responsibility of organizations and providers, supported by resources, policies, and systems, to shift services at the point of care, rather than people having to work around services and their arcane rules to get what they need. The primary question examined in this critical narrative review, therefore, was as follows: what interventions are effective in preventing IPV and its impacts, and how can bringing an equity-oriented, trauma- and violence-informed lens enhance intervention research and practice?

## Method

Following the method outlined in Scott-Storey et al. [49] examining a related and similarly complex topic (men’s experiences of IPV), we undertook a critical narrative literature review of the current state of knowledge on how equity-oriented concepts, especially TIP and more specifically TVIC, have been applied in research and practice interventions for IPV. Knowing from previous reviews [43•] that the existing evidence on TVIC and IPV is heterogenous and diffuse, we did not use traditional systematic review methods, but rather conducted a focused search (in terms of concepts) but in a relatively broad range of literature and evidence sources. We looked for peer-reviewed qualitative, quantitative, and mixed-method articles, theoretical papers, and high-quality gray literature (e.g., government documents, research institute reports) across multiple databases up to July 2022 (Cochrane, Campbell, MEDLINE via PubMed, Scopus for a consolidated search, Embase, and PsycINFO; Appendix), with a focus on systematic and other reviews. We then ran a focused Google Scholar search to identify additional key items and relevant gray literature, including government documents. Literature was limited to English and had a beginning date of 2017, unless the most recent systematic review preceded this date. Key search terms included “intimate partner violence” and “domestic violence” combined in various ways with “trauma-informed” and “trauma- and violence-informed.” In total, these searches identified 227 unique articles with varying degrees of relevance to our core topics, i.e., they were principally about providing care or services to IPV survivors and included the concept of trauma (and violence)-informed care/practice, with the addition of the violence concept meaning the approach explicitly attended to forms of structural inequities, including sexism, racism, ableism, and other forms of discrimination and/or stigma. Additional reference chaining identified a key sub-set of studies that include TVIC as a component of a larger IPV-focused or IPV-related complex intervention. These results were situated in a high-level summary of the latest evidence, drawn from recent (i.e., those most recently available in each intervention domain) systematic reviews of IPV interventions in various domains. Using a critical perspective to evaluate and synthesize the literature, we do not present quantitative details about these articles.

## Findings

The review findings are presented as a narrative synthesis of evidence according to specific types of interventions for preventing occurrence, recurrence, or sequelae of IPV. Findings from systematic reviews are prioritized, with general effectiveness summarized first, followed by a discussion of whether/how TIP or TVIC components have been addressed for each intervention domain.

### Identifying IPV

Evidence-based guidelines generally do not advocate routine, universal IPV screening, with multiple trials showing that while screening can increase IPV disclosures, these alone are not linked to additional referral to services, or benefits to those who disclose [50•]. Screening approaches, which rely on structured questions asked at intake or even by computerized assessment, are not trauma- and violence-informed [51]. Rather, most guidelines emphasize asking about IPV using a case-finding approach grounded in knowledge of clinical indicators and risk factors [52]. We have recently argued that adding explicit attention to TVIC principles to the WHO’s LIVES (Listen, Inquire, Validate, Ensure Safety, Support) Protocol [52] meets the criteria for an evidence-based, trauma- and violence-informed approach to IPV identification and referral [51].

### Counseling Interventions

Once IPV survivors are identified, there is some evidence that brief counseling interventions to provide immediate support and stabilization for women experiencing IPV can be effective. A systematic review and meta-analysis of 21 studies shows a large overall effect size of 1.02 (calculated by the authors as a 34% benefit of engaging in intervention, across studies), especially in mental health and life functioning outcomes, with moderate effects on safety, violence, and substance use outcomes. These results were largely driven by individual-level interventions, especially cognitive-behavioral therapy (CBT) tailored for IPV survivors, and by interventions delivered one-on-one versus in groups. Of note, interventions specifically adapted to address IPV experiences showed the greatest benefit, whether delivered in shelters, or in the community [53•]. These findings, while still requiring replication and follow-up studies, reinforce the need for tailoring to both the IPV context and women’s unique experiences; however, from a TVIC and equity perspective, individualized psychological therapy is inaccessible to many survivors, due to out-of-pocket costs, local availability (especially in non-urban areas), and/or wait-lists, and generally does not address structural inequities.

### Advocacy-Based Interventions

A Cochrane systematic evidence review [54•] found some trial-level evidence for efficacy of advocacy-based interventions for women using shelters and/or facing greater barriers to services due to more severe violence experiences or socio-economic marginalization. A more recent scoping review of both qualitative and quantitative studies [55•] examined social support, including advocacy-based interventions, and differentiated those that focused specifically on the individual survivors’ needs, from those that included the survivor and their broader social/community network, finding benefits from most interventions in terms of women’s mental well-being, while calling for more robust methods. Of note, both reviews highlight the need for interventions that are grounded in theory, an ecological understanding of IPV alongside the intersection of survivor needs and structural factors, and a woman-centered approach to safety that does not assume that leaving a relationship is safer, nor is what all women want. Thus, the interventions identified as most promising in this domain, as with the counseling interventions above, are those that best align with a TVIC approach.

### Shelters and Other Forms of Housing

While it is widely recognized that emergency shelters, second stage/transitional, and other forms of safe housing are essential for women and their children at high risk of injury or death due to IPV, systematic reviews [56•, 57] find limited, and relatively low-quality, research evaluating the effectiveness of these services. From an equity/TVIC perspective, the most important consideration in this domain is a system-level approach to making safe and affordable housing available, on a priority basis, to women and children experiencing IPV.

### Technology-Mediated Interventions

A systematic review of 25 studies examining technology-mediated interventions for IPV found wide variability in types of technology, and focus of intervention, with most designed to support either primary prevention via educational content or identification through screening [58•]. Those focusing on specific outcomes, especially safety, mental health, or use of services/supports, showed the most benefit overall. While simple interventions, such as short scripts, are appealing for their ease of integration into services, this strategy when used alone is not aligned with a TVIC approach [59], nor is asking women to self-screen for IPV on a device. While moderately complex interventions that add standard IPV resources (videos, text messages, and audio) are useful alternatives to print-based resources [60], more complex technology-based interventions that provide tailored, interactive safety and health strategies and needs-based access to real-time supports were the most effective in experimental studies at improving health and safety, and reducing decisional conflict overall, or among specific groups of women [61, 62, 63]. With the efficacy of technology-mediated IPV interventions that are theoretically grounded and woman-centered becoming established for community samples, refinements to meet the needs of women facing intersecting forms of oppression are being developed and scaled [64]. Continued focus on ensuring that TVIC principles are embedded in these interventions to support women’s emotional, physical, and cultural safety, and their choice and autonomy, while building on their existing strengths, is crucial to their ongoing effectiveness and utility [62].

### Interventions for Couples and Perpetrators

The evidence regarding interventions for couples where IPV is present is generally weak, with systematic reviews indicating high variability in design, few comparative studies of generally lower quality, and mixed results [65•, 66, 67]. A key consideration is ensuring that the intervention does not lead to additional harms to the survivor; thus, studies generally showing some benefit are those in which the violence is situational and/or bi-directional, or in which other related issues, such as substance use, are a key factor. In cases of coercive control/intimate terrorism, couples’ therapy is not recommended and, from a TVIC perspective, would not be considered a safe option. Clinically, this is an important consideration, as markers for intimate terrorism are more likely to be present in those seeking services than in broader community samples [68•].

Three recent systematic reviews find mixed results among studies examining interventions for men perpetrating IPV [69•, 70•, 71], with the general finding that higher quality, comparative studies are less likely to demonstrate that group-based interventions (the primary delivery model) reduce IPV. The generally poor methods used in these studies make it difficult to know whether theoretically grounded interventions that disrupt men’s conceptions of power and control (e.g., the Duluth model) are more or less successful than cognitive-emotional models that focus on behavior change, anger regulation, etc. As noted in the two realist reviews of these kinds of intervention studies [70•, 71], not enough of them actually define and describe the theoretical mechanism of action to draw firm conclusions.

In the face of a largely inconsistent evidence base, what can a TVIC lens add? More recent studies, as part of an intervention model (e.g., CBT, Duluth), examine how IPV is linked, contextually and sometimes even causally, to the perpetrator’s own trauma experiences, and/or their cognitive/emotional processing ability [72–81]. In fact, what emerges from a close reading of this literature is that “trauma-informed” in this context focuses exclusively on how trauma experiences impact psychological processes such as emotion regulation, substance use, and attachment and can be inferred as “causing” perpetration, leading to trauma-specific approaches to healing perpetrators such that they stop using violence. However, this approach to perpetrator intervention has yet to show effectiveness. From a critical, equity-oriented, and trauma- and violence-informed stance, this kind of rationale must be approached cautiously, as it reinforces individual-level factors and intervention approaches that may exonerate perpetrators rather than situate their behaviors, and accountability, in the social-ecological framework, where both individual and collective accountability and action are required.

## Discussion

### What Makes IPV Interventions Successful?

In summary, existing evidence for IPV interventions targeting secondary and tertiary prevention remains heterogenous and generally situated at the individual level of the ecological framework, i.e., supporting survivors in preventing recurrence and/or addressing the specific effects of IPV, or perpetrators or couples in not using violence. IPV interventions shown to be most effective, or indicating promise of effectiveness, tend to be those that understand the complexity of IPV as rooted in factors from across the social ecology, especially the patriarchal norms and practices that enable gendered violence, and acknowledge that these factors intersect, meaning more risk and fewer supports for some, and more resilience and help for others. In a realist review of 60 reviews examining psychosocial IPV interventions, Paphitis and colleagues [82••] focus on mechanisms of intervention, including both the resources provided to survivors, but also the reasoning that underpins the theory of change—i.e., how survivors can reframe their experiences and behaviors to find emotional safety and well-being, and freedom from violence. Beginning with the recognition that IPV is a complex phenomenon requiring complex interventions, they analyze existing evidence using a context, mechanisms, and outcomes (CMO) approach, and then integrate their findings with expert input. They reinforce what we see in the above summaries of existing evidence, specifically that “interventions that are individually adapted, IPV-tailored, and trauma-informed are likely to yield the best results” (p. 22). They further emphasize the importance of community buy-in and partnerships, and culturally safe and appropriate approaches with a deep understanding of the context in which the intervention is delivered. These framing values, a “multi-layered” approach to understanding IPV and its diverse effects on health and well-being, combined with the recognition of its co-occurrence with other complex issues (including substance use, infectious diseases, and, importantly, structural violence such as poverty, lack of safe housing, racism, and ableism), support successful implementation and outcomes. A lack of attention to these issues can not only mean less effective and/or ineffective interventions, but can be actively harmful, leading to increased inequities and exclusion of certain groups, erosion of trust in providers and organizations (and therefore less help-seeking), and increased harm for survivors and communities.

An ecological approach also aligns with emerging evidence in primary prevention of IPV, especially in lower income settings, where community-level interventions using complex designs specific to gender roles and family well-being are showing promise [83••], as are structural-level interventions [84••] including enhancing women’s economic empowerment [85••]. Primary prevention work in higher income settings, which tends to be more individually targeted, shows mixed results for a range of strategies, but some promise for those that include teaching younger people about healthy relationships and social-emotional skills to prevent behaviors linked to later IPV [86•].

As we move to more complex, trauma- and violence-informed intervention development and evaluation, conceptual clarity is required. A key concern noted above is how the concept of “trauma-informed” is brought into interventions. In those targeting either secondary prevention through work with perpetrators or tertiary prevention through addressing impact of IPV on survivors, there is conceptual slippage from “trauma-informed” to “trauma-specific”—i.e., treating (e.g., through CBT) past or current trauma as the cause/consequence of the violence [87•, 88]. In work with perpetrators, this can elide a structural analysis that grounds their behaviors, and indeed their own trauma experiences, in structural factors. In work with survivors, a focus on individual trauma may lead to interventions that do not account for the multi-layering and complexity required for success [41, 42].

An explicit focus on TVIC, which integrates a critical and structural analysis into care principles and practices, is conceptually grounded in a social-ecological understanding of health and well-being and aligns extremely well with the complexity approach to IPV intervention supported by existing evidence and reviewed above [82••, 89•]. TVIC, as we have constructed and tested it, is a core component of equity-oriented care [47••, 90], which is itself showing promise in improving care interactions and health outcomes [91, 92]. Applied in complex IPV interventions, TVIC is a way to ensure provider education and practice are both safe and structurally competent [62, 93, 94, 95]. It is also showing promise in related areas of practice [6].

### Implications for Research, Practice, and Policy

As we have seen, interventions that account for complexity across several domains, including recognizing (1) the causes of IPV as interactive across the social ecology, but rooted in gendered norms about power; (2) IPV as an experience with multiple and variable impacts that often co-occurs with other complex health and social problems, both individual and structural; and (3) sites of intervention, whether individual practices, organizations, or communities, as complex adaptive systems [96], are what is needed to advance the field. In sum, people live complex lives, with their well-being often shaped as much (or more) by social and structural factors arising from their intersecting social locations, as from their individual behaviors. When this complexity includes IPV, the effects are also multidimensional, compounding, and complex, and a survivor’s access to support is shaped by many of the same factors noted above. From a practice perspective, a key challenge is resourcing and sustaining what can be expensive, multi-component interventions [82••].

In policy, a TVIC approach means resourcing IPV programs and services such that they can address complexity and be sustained, ideally by embedding them in relevant existing systems of care. For example, the IPV intervention developed by Jack and colleagues is now embedded in the Nurse-Family Partnership® (NFP) intervention [93, 97]; related resources, funded by a government ministry, support nurse home visiting practice in Canada [98]. Given the international reach of the NFP program, this approach to integration of a TVIC IPV component can serve as a model. The key drivers of such an integration were the NFP’s approach to continuous, evidence-based improvement, and the values alignment between NFP’s foundational program principles and the TVIC principles, especially that the client (generally a very young woman facing various kinds of marginalization) and child are the center of the intervention, and the nurse develops and sustains a relationship built on trust, respect, choice, and the woman’s strengths, while prioritizing her emotional, physical, and cultural safety, including her privacy. The flexibility afforded the nurses as clinical specialists also allows for decisions in the intervention process that prioritize these principles; for example, rather than a formal, structured assessment tool, nurses use a life history timeline that is collaboratively developed with the client, giving her control over the narrative (e.g., what to share, where to start), identifying historical events in her life, for example, early traumas that might shape her current situation, as well as events that contributed to strength and resilience (S. Jack, pers. comm.). However, this kind of approach to adapting existing, and developing new, system-level interventions that are trauma- and violence-informed also means upstream attention to the aspects of the social ecology that frame IPV causes and consequences, including anti-discrimination, anti-poverty and affordable housing policies, and accountability for perpetrators throughout the legal, social, and health care systems.

For intervention designers and evaluators/researchers, careful attention to rigourous research design is required, using multi- and mixed-method approaches that can address complexity [99], and outcome measures that properly frame and assess IPV as a gendered and patterned phenomenon, and its impacts as multidimensional, without devolving to IPV recurrence as the sole primary outcome, for example, in evaluations of survivor interventions [19, 20•, 21•]. The field of IPV research has produced many null trials that did not properly account for complexity in their design and implementation, and recent reviews indicate that many of the lessons from these trials go unlearned as these designs are replicated to present day. Specifically, trial designs often exclude “outlier” or otherwise diverse (complex) participants and communities. While including diversity and attending to the roots of structural inequities that perpetuate IPV have been found to increase the unpredictability of results [100], without such attention, existing interventions time and again show limited effectiveness and low acceptability to survivors, and in some cases do more harm than good. Bringing an equity-oriented, trauma- and violence-informed lens to intervention work overcomes these barriers and creates the space for real change, in both policy and practice, to prevent IPV and its impacts.

This review is an initial examination of how TVIC, with a particular focus on structural forms of violence and the social level of the ecological framework, can enhance our thinking about the design and delivery of IPV interventions. The review was limited by a focus on English language and primarily peer-reviewed publications, and by a paucity of research in some areas. Intervention development and research that integrates more structural elements with a focus on primary prevention is urgently needed; this is especially true in higher income settings, which have lagged behind lower income settings in this regard.

## Conclusion

Interventions to prevent and mitigate the effects of IPV must include an understanding of the circumstances of people’s lives, and how this, and related forms of violence are rooted in social-structural factors, which intersect across the social ecology. We must acknowledge that structural forms of violence filter down to everyday experiences, including interactions with legal, health, and social services. Viewed this way, people’s responses to trauma and violence, including substance use and poor mental health, are predictable consequences of threatening events, which can include their everyday experiences of stigma, discrimination, judgement, and poor or dismissive service. This is especially the case when inequities and system-induced trauma are ongoing. Bringing a trauma- and violence-informed, equity-oriented approach to IPV interventions across the prevention spectrum presents a major evolution in IPV research, practice, and policy.

## Appendix. Primary search terms

### PubMed

((domestic violence[majr] NOT child abuse[mesh] NOT elder abuse[mesh]) OR “intimate partner violence” OR “partner violence” OR battered women[majr]) AND (“trauma-informed” OR “trauma- and violence-informed”).

### Scopus

(TITLE-ABS-KEY (“domestic violence”) OR TITLE-ABS-KEY (“intimate partner violence”) OR TITLE-ABS-KEY (“partner violence”) AND TITLE-ABS-KEY (“trauma-informed”) OR TITLE-ABS-KEY (“trauma- and violence-informed”)) AND (LIMIT-TO (PUBYEAR, 2022) OR LIMIT-TO (PUBYEAR, 2021) OR LIMIT-TO (PUBYEAR, 2020) OR LIMIT-TO (PUBYEAR, 2019) OR LIMIT-TO (PUBYEAR, 2018)) AND (LIMIT-TO (DOCTYPE, “ar”) OR LIMIT-TO (DOCTYPE, “re”)).

### EMBASE (map to subject headings)

1. (domestic violence or intimate partner violence or partner violence).mp. [mp = title, abstract, heading word, drug trade name, original title, device manufacturer, drug manufacturer, device trade name, keyword heading word, floating subheading word, candidate term word]
2. limit 1 to yr = “2017 -Current”
3. (trauma-informed or trauma and violence informed).mp. [mp = title, abstract, heading word, drug trade name, original title, device manufacturer, drug manufacturer, device trade name, keyword heading word, floating subheading word, candidate term word]
4. 2 and 3

### PsycINFO

MAINSUBJECT.EXACT(“Trauma-Informed Care”) AND MAINSUBJECT.EXACT(“Intimate Partner Violence”).

## Google Scholar

(“domestic violence” OR “intimate partner violence”) AND (“trauma- and violence-informed”).

The terms above were used in each specified database to capture any paper that addressed both IPV and TIP/TVIC. Additional focused searches were conducted in each source for systematic reviews of IPV interventions by combining the IPV indexing term(s) and the keyword “intervention” with the DB-specific indexing term for “systematic review.” In the Cochrane and Campbell systematic review databases, we searched for “violence” and reviewed all results for relevance. Inclusion dates for these results were reviewed by hand. For example, for PubMed, the search was:

intimate partner violence[majr] AND systematic review AND intervention.
